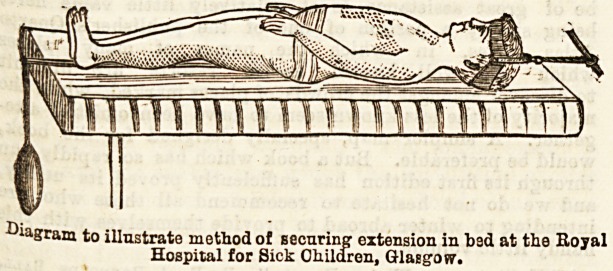# Spinal Caries in Sick Children, Diagnosis and Treatment

**Published:** 1893-02-11

**Authors:** 


### ROYAL HOSPITAL FOR SICK CHILDREN, GLASGOW.

### SPINAL CARIES IN CHILDREN. DIAGNOSIS AND TREATMENT.

The diagnosis of caries of the spine of children is by no means easy in the early stage, and much harm undoubtedly results from delay in diagnosis till the typical curvature develops with its attendant evils, and when treatment has to be directed, not only to the original disease, but also to such secondary complications as paraplegia and those due to the displacement and compression of the thoracic and abdominal viscera.

Early diagnosis is as essential for the treatment of this disease as it is in similar affections of the joints or elsewhere. The physician who waits till the lung has broken down from tubercular disease ere treatment is commenced, can expect but a poor result.

The points worthy of attention for early diagnosis may be grouped as follows: 1. Altered disposition. 2. Rigidity of trunk. 3. Pain, (a) in back, (b) peripheral. 4. Paralytic symptoms. 5. Family history. The later consequences are the angular curvature as described by Potts, and abscess formation. In children the arrest of the usual active movements, a tendency to keep quiet and in one position, and to lean against the furniture, often to support the chin on the edge of the table or elsewhere, or to remain for prolonged periods in the recumbent posture, the assumption of a grave and old man like attitude, with a steady soldier like gait, not infrequently leading the parents to express admiration of the square shoulders of the child and its general good behaviour. The child frets and cries when compelled to move, otherwise, is usually quiet and good, often with an unusual mental brightness.

The gait and attitude are the result of muscular rigidity, active here as elsewhere, in the fixation of a diseased and painful part. All the muscles of the trunk are affected, and the more powerful dorsal group cause the shoulders to be thrown back. The condition of the muscles is evident to sight and palpation, and is a point of considerable importance in differential diagnosis, as for example in the antero posterior curvature of Rachitis, in which a noticeable feature is the complete muscular relaxation.

Pain is rarely in the earlier stages complained of as being in the back, more frequently is referred to the peripheral distribution of some spinal nerve, as along the great occipital nerve in atlanto-axoid disease, along the arm or intercostal in cervico dorsal, and most frequent of all the very common "belly ache" in lower dorsal affections, while "growing pains" in the legs may be really due to an irritation at the nerve roots, which may also give rise to a certain amount of motor disturbance indicated by unsteadiness of gait, with a constant tendency to fall. On attempting to rise, the rigidity is specially notable, the child rests the hand on the knee or grasps some article of furniture, and so levers itself up instead of arching the spine as usual.

Pain in the back may be elicited by local pressure, or jarring the spine through the head. The use of a hot or cold sponge used alternately is also effective in many cases.

Paralytic symptoms may be due to several causes. In the early stage of the disease, usually from congestion or inflammatory effusion interfering with the nerve roots, or to congestion of a segment of the cord corresponding to the portion of the spine affected. In the later stages, compression of the cord from cicatricial tissue or bone displacement may lead to paralysis of a more serious nature, amenable only to surgical interference. The formation of Potts' curvature by the bending forward of the spine from the destruction of one or more vertebral bodies, is a natural result if not arrested by prompt preventive measures.

Abscess formation in connection with caries of the spine is neither a constant nor necessary corollary, and is in many cases due to the want of treatment.

As in other tubercular affections, suppuration is a something superadded to, and not a necessary part of the original disease. According to the part of the spine affected, the pus when formed finds its way to the surface in different situations, guided thereto by fascias, by muscles, or along the areolar tissue spaces round blood vessels and nerves. On the other hand, the pus may accumulate round the site of the diseased bones as in the posterior mediastinum, or behind the pharynx leading to difficulty in swallowing, or respiratory trouble.

Treatment consists in, 1. Fixation of the spine.

2. Attention to general health. 3. Treatment of secondary symptoms as they arise. The fixation is the point on which most stress is laid, and the more perfect this is, the better are the prospects of treatment. The difficulties of securing complete fixation of the mobile spine are apparent when we consider the inclusion by the retentive apparatus of the ever-changing bulk of the chest and abdomen.

In the Royal Hospital for Sick Children, a large number of cases are constantly under treatment, both in the out-door department and in the wards where the disease is generally in an advanced stage, requiring treatment for paraplegia, or abscess formation. The out-door cases are treated in one or both of two ways. 1. Rest in bed with fixation apparatus. 2. Use of jackets after Sayre's method. In regard to the bed treatment, the nurses in connection with the surgical department visit the homes of the children and arrange the bed. Many of the homes are miserably poor, and in such an orange box, which may be got for a few coppers, answers admirably, limiting as it does lateral movement, and affording, by means of holes bored in the ends, a ready means of applying extension and counter extension made for the head and pelvis. The head is caught by a belt passed round the forehead under the frontal eminences, and under the occipital protuberances behind. To this belt are attached on both sides above the ears straps, which are fixed to the top of the bed, with the intervention of a strong elastic band.

Diagram to illustrate method of securing extension in bed at the Royal Hospital for Sick Children, Glasgow.

Counter extension is made by means of a pelvic girdle, as by a soft towel placed under the illiac crests, and stitched opposite the sacrum, from which point traction is made by weight and pully over the foot of the bed. The extending force may be aided by elevation of the top of the bed, so that the body may tend to drag from the head. The head band is well borne, and does not readily slip off. It ought to be about three-quarters of an inch in width, and made of leather, or several layers of strong canvas, lined with flannel.

If the upper part of the spine be affected, sand bags placed under and on each side of the neck are invaluable. They must be accurately made, and moulded to fit the sinuous outlines of the neck. When the disease is in the dorsal or limba region, a light plaster jacket is used in addition if the child is restless and difficult to control.

Even in comparatively squalid homes it is found possible to carry out these measures, which, if accompanied with a suitable dietary, attention to ventilation of the room, and cleanliness, lead to an early improvement in the general condition of the body, so that in six or eight months, when pain has ceased and the progress of the disease is stayed, a suitable jacket may be applied, and the upright position resumed. When practicable, great good may be got in the direction of improved nourishment, by massage applied to the limbs twice a day for five to ten minutes. The jacket in use is the plaster of Paris jacket of Sayre.

The child is suspended by a pulley, acting through slings placed under the armpits, and a head bridle catching under the occipit and chin, till the toes alone touch the table. A specially woven flannelette vest is then drawn over the body from below (this flannelette is woven as a continuous tube, from which a sufficient length is cut off as required), so as to extend from below the trochanters and from above the shoulders, where it is pinned on each side of the neck to secure it in position. A pad of cotton-wool, 4in. by 3in., is placed under this in front of the abdomen, also thin pads over the crests of the illium, and by the sides of the affected portion of the spine. The plaster of Paris bandages are now applied and firmly pressed, but not pulled against the body. The margins of the jacket are best formed by applying a few turns of the bandage edgewise to secure a proper thickness and regularity. Under each armpit a roll of cotton-wool is incorporated with the first turns of bandage. The margins having been formed, the rest of the bandages, seven or eight in number, are applied to the intervening space incorporating several willow laths—one in each mid axillary line, and one on each side of the spine—and the jacket finished by turning up and down the excess of the flannelette, and coating the whole surface with plaster paste, and as this is about to set, applying the hand freely moistened with water to give a smooth skin to the jacket. The lower edge of the jacket ought to be midway between the crest of the illium and the trochanters of the femur, slightly bevelled upwards opposite the thighs in front. The upper edge ought to pass well up into the armpits, behind as far as possible upwards between the shoulders, and towards the manubrium sterni in front. Thus not only is the body held rigid, but the jacket carries the weight of the arms and shoulders on the crests of the illium. Such a jacket applied to the skin, carefully cleansed and disinfected by a dusting of boracic powder, may be worn for months, until, in fact, it becomes too small. Other and more expensive apparatus such as poroplastic jackets, and the other complicated mechanical supports are now never used.

The complications which most requires active interference is abscess formation. The history of a spinal case when the abscess has been allowed to burst or been opened by a surgeon, and become septic is sad in the extreme, the progress of the case attended with much pain and suffering to the patient, trouble, worry and disappointment to those who attend. The avoidance of sepsis is the one guiding principle. In the following is the method now usually employed.

The skin over and around the side of the proposed opening is thoroughly cleansed with soap and water, with turpentine and methylated spirits, and disinfected with 1-20 solution of carbolic acid. An opening is then made about one inch in length through a fairly healthy skin area, the pus evacuated and thoroughly washed out by means of an irrigator, with 1-4000 sol. of perchloride of mercury. The interior of the cavity is then scraped by a Volkmann's spoon, and irrigation continued till the fluid issues from the wound comparatively clear. The cavity being then emptied, the wound is accurately stitched with the view of securing immediate union, that fertile source of wound infection, the drainage tube, not being used. Within the next twenty-four hours a considerable reaccumulation of serous fluid occurs, and this is got rid of by aspiration. In this way in dispensary practice it is found possible to keep such cases free from sepsis thus permitting the case to progress in its natural course, without the addition of septicæmia and its hectic fever, which effectually counteract any measures taken to raise the general health of the individual, and the vitality of the tissue by which alone the tubercular diseases of the spine may be combated.

Should subsequent reaccumulation occur the same measures are adopted, but in many cases such does not take place, provided a thorough fixation scheme be carried out, and means used to improve the general body condition.

Medicinal treatment consists principally in the use of cod liver oil—alone or combined with malt preparations. The use of the tonic syrup preparations are less favoured from their tendency to induce gastric disturbance.

The treatment of paralytic symptoms, when due to congestion or irritation of nerve roots, is that of the general condition already described. Provided the rest be absolute, rapid recovery of the muscular power may be expected, provided always there be no septic irritation at the seat of mischief. The actual cautery and other forms of counter-irritants are seldom used.

When the paralysis does not disappear after a reasonable period of fixation, recourse is had to operative interference in the form of laminectomy, *i.e.*, the removal of the spines and laminæ over the affected area, and the liberation of the cord from its compressing agent. Narrowing of the spinal canal is not common, but granulation or cicatrical tissue may exert injurious pressure on the cord, and such can be readily relieved with marked benefit. Even while the cord is exposed, after the relief of the constriction, the gradual return of the pulsation will indicate a favourable issue to the case.

## Figures and Tables

**Figure f1:**